# Genomic Diversity of Enterotoxigenic Strains of *Bacteroides fragilis*

**DOI:** 10.1371/journal.pone.0158171

**Published:** 2016-06-27

**Authors:** Jessica V. Pierce, Harris D. Bernstein

**Affiliations:** Genetics and Biochemistry Branch, National Institute for Diabetes and Digestive and Kidney Disease, National Institutes of Health, Bethesda, MD, 20892, United States of America; University of Ulster, UNITED KINGDOM

## Abstract

Enterotoxigenic (ETBF) strains of *Bacteroides fragilis* are the subset of strains that secrete a toxin called fragilysin (Bft). Although ETBF strains are known to cause diarrheal disease and have recently been associated with colorectal cancer, they have not been well characterized. By sequencing the complete genome of four ETBF strains, we found that these strains exhibit considerable variation at the genomic level. Only a small number of genes that are located primarily in the Bft pathogenicity island (BFT PAI) and the flanking CTn86 conjugative transposon are conserved in all four strains and a fifth strain whose genome was previously sequenced. Interestingly, phylogenetic analysis strongly suggests that the BFT PAI was acquired by non-toxigenic (NTBF) strains multiple times during the course of evolution. At the phenotypic level, we found that the ETBF strains were less fit than the NTBF strain NCTC 9343 and were susceptible to a growth-inhibitory protein that it produces. The ETBF strains also showed a greater tendency to form biofilms, which may promote tumor formation, than NTBF strains. Although the genomic diversity of ETBF strains raises the possibility that they vary in their pathogenicity, our experimental results also suggest that they share common properties that are conferred by different combinations of non-universal genetic elements.

## Introduction

Organisms that belong to the genus *Bacteroides* are the most abundant Gram-negative bacteria in the human gut microbiome. These bacteria are thought to play important roles in the development of the immune system, gut homeostasis, and metabolism [1, 2]. Like other members of the genus, *B*. *fragilis* is a commensal organism, but it is unique in that it is also associated with disease. This species of *Bacteroides* is the most commonly isolated anaerobic bacteria in abdominal infections and sepsis. A subset of *B*. *fragilis* strains known as enterotoxigenic or ETBF strains cause diarrhea. ETBF strains produce a metalloprotease called fragilysin (Bft) that cleaves E-cadherin *in vivo* and a variety of other proteins including type IV collagen and fibrinogen *in vitro* [3, 4]. The toxin is encoded in a pathogenicity island (BFT PAI) that is acquired via horizontal transfer and that is part of a larger conjugative transposon known as CTn86 [5]. Interestingly, ETBF strains have increasingly been associated with colorectal cancer. Mono-colonization of mice that harbor a mutation in the adenomatous polyposis coli (APC) gene with an ETBF strain leads to high levels of fragilysin-dependent tumor formation in the gut [6]. The finding that the *bft* gene is more highly associated with colon mucosal samples obtained from colorectal cancer patients than from healthy controls suggests that exposure to Bft is a risk factor for the development of malignancies [7]. By cleaving E-cadherin Bft likely activates the β-catenin signaling pathway, which is altered in many neoplasms [8]. There is also evidence that Bft may promote tumor formation by eliciting high levels of reactive oxygen species that damage host cell DNA [9]. ETBF strains appear to be quite widespread, and as many as 30% of all individuals may carry these strains asymptomatically [10].

Although the synthesis of Bft presumably enhances the fitness of ETBF strains in the gut, the success of these strains must also depend on their ability to utilize available nutritional resources, to interact productively with host cells, and to compete effectively with other bacteria. The survival strategies that ETBF strains employ are unclear, however, because these strains have not been well characterized. BTF PAIs from a few ETBF strains have been sequenced [11, 12], but the complete genome sequence of only one ETBF strain has been reported [13]. Our current understanding of *B*. *fragilis* biology is derived almost entirely from studies on nontoxigenic clinical isolates (NTBF strains) that have served as a reference. Predictions based on the genome sequences of NTBF strains as well as direct experimental analysis has indicated that they have the ability to metabolize a wide array of the complex carbohydrates found in the gut [14–16]. The NTBF strain NCTC 9343 produces a symbiosis factor (polysaccharide A) that signals through Toll-like receptor 2 to suppress the immune response [1]. The same strain produces so-called “commensal colonization factors” that facilitate colonization of specific niches within the gut that are distinct from those occupied by other bacteria [17]. To prevent the growth of other related organisms, NTBF strains also secrete antimicrobial proteins. For example, it was recently shown that strain 638R packages a perforin-like protein into outer membrane (OM) vesicles to kill other NTBF strains [18]. At least some NTBF strains produce type VI secretion systems (T6SSs), which are used by a wide range of Gram negative bacteria to secrete effector proteins that target other bacteria as well as host cells [19, 20]. Multiple effector/immunity proteins associated with the T6SS have now been identified [21] and strains with the T6SS are antagonistic to other *Bacteroidales* both *in vitro* and in the mammalian gut [22, 23]. Available evidence suggests that T6SS loci may be spread via horizontal gene transfer among microbiome-associated bacteria [24].

The survival of ETBF strains in the gut may also be influenced by their ability to form biofilms. Strain NCTC 9343 forms weak biofilms in rich media, but biofilm production rises in the presence of bile salts [25]. Interestingly, the addition of bile salts leads to a concomitant increase in the synthesis of fimbriae-like appendages and drug efflux pumps. This observation suggests that biofilm formation facilitates adhesion and antibiotic resistance, which in turn might enhance survival. In addition, the transcriptional profile of *B*. *thetaiotamicron* biofilms resembles that of cells residing in the gut more faithfully than planktonic cells [26]. The expression of genes involved in polysaccharide utilization and capsule biosynthesis, which may aid survival in the gut, is upregulated in biofilms. Finally, the formation of biofilms may not only alter the physiology of ETBF strains, but also their ability to induce disease by promoting growth on epithelial surfaces and preventing the diffusion of Bft. Indeed, biofilms are thought to increase inflammation and have been associated with human colorectal tumor samples [27].

In this study we sought to obtain insights into the relationship between ETBF and NTBF strains and to determine whether ETBF strains might have common features that enable them to colonize the gut and cause disease. To this end, we obtained draft genome sequences of four different ETBF strains. Sequence analysis not only revealed striking genomic diversity among the strains, but also indicated that ETBF strains arose more than once through the independent integration of the BFT PAI and flanking CTn86 sequences into the *B*. *fragilis* chromosome. Although only a few genes that reside primarily in the BFT PAI or CTn86 were identified in all four strains and the previously sequenced strain, a larger set of genes was identified in four of the five strains. Despite the sequence diversity, we found that several ETBF strains were sensitive to an anti-microbial protein secreted by NTBF strain NCTC 9343 and had a greater ability to form biofilms than the NTBF strain. Taken together, our results suggest that while ETBF strains are more diverse than previously suspected, they nevertheless share several common phenotypic properties.

## Results

### Genome analysis reveals considerable diversity among *B*. *fragilis* ETBF genomes

**Fig 1:**
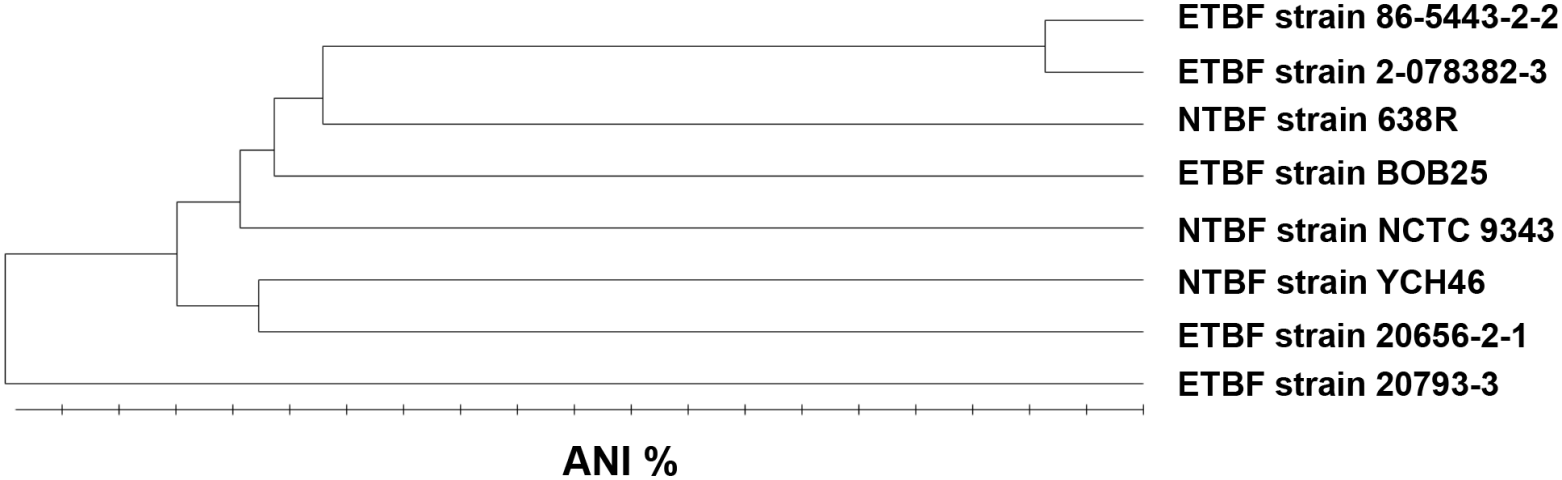
Phylogeny of *B*. *fragilis* ETBF and NTBF strains. Evolutionary distance values are based on average nucleotide identity (ANI) as determined by CLGenomics software. The scale ranges from 99–100%.

To determine the degree of similarity between NTBF and ETBF strains of *B*. *fragilis*, we produced draft genome sequences of ETBF strains 2-078382-3, 20793–3, 20656-2-1 and 86-5443-2-2 using Illumina paired-end next generation sequencing (Table 1). The first three strains were isolated from humans in the United States who presented with diarrheal disease [28], while strain 86-5443-2-2 was isolated from a porcine intestinal infection [29]. Phylogenetic analysis of these strains, along with a newly sequenced ETBF isolate BOB25 [13] using average nucleotide identity (ANI) as a measure of relatedness [30], showed that ETBF strains do not co-cluster as a specific group within the larger family of *B*. *fragilis* strains (Fig 1). The BFT PAI was therefore likely acquired independently by distantly related *B*. *fragilis* strains. Curiously, the genomes of the human isolate 2-078382-3 and the piglet isolate 86-5443-2-2 are closely related. This finding suggests that similar sets of genes may be required for colonization of the human and porcine colon.

**Table 1:** Summary of draft genome sequences of ETBF strains performed using Illumina paired-end whole genome sequencing.

Strain	Source	Genome size	GC ratio	CDS	rRNA	tRNA	Plasmid	Contigs	N50	Fold coverage
2-078382-3	Infant DiarrheaUSA	5,212,728	43.12	4,429	7	63	Included Contig 77 pBFP53	143	346,660	>50
20793–3	Infant Diarrhea USA	5,217,860	43.18	4,492	3	63	None	52	301,080	>400
20656-2-1	Adult Diarrhea USA	4,902,607	43.46	4,144	3	61	Included Contig 63 pBI143	67	176,326	>400
86-5443-2-2	Piglet	5,258,210	43.34	4,665	3	61	None	156	181,621	>100

**Fig 2:**
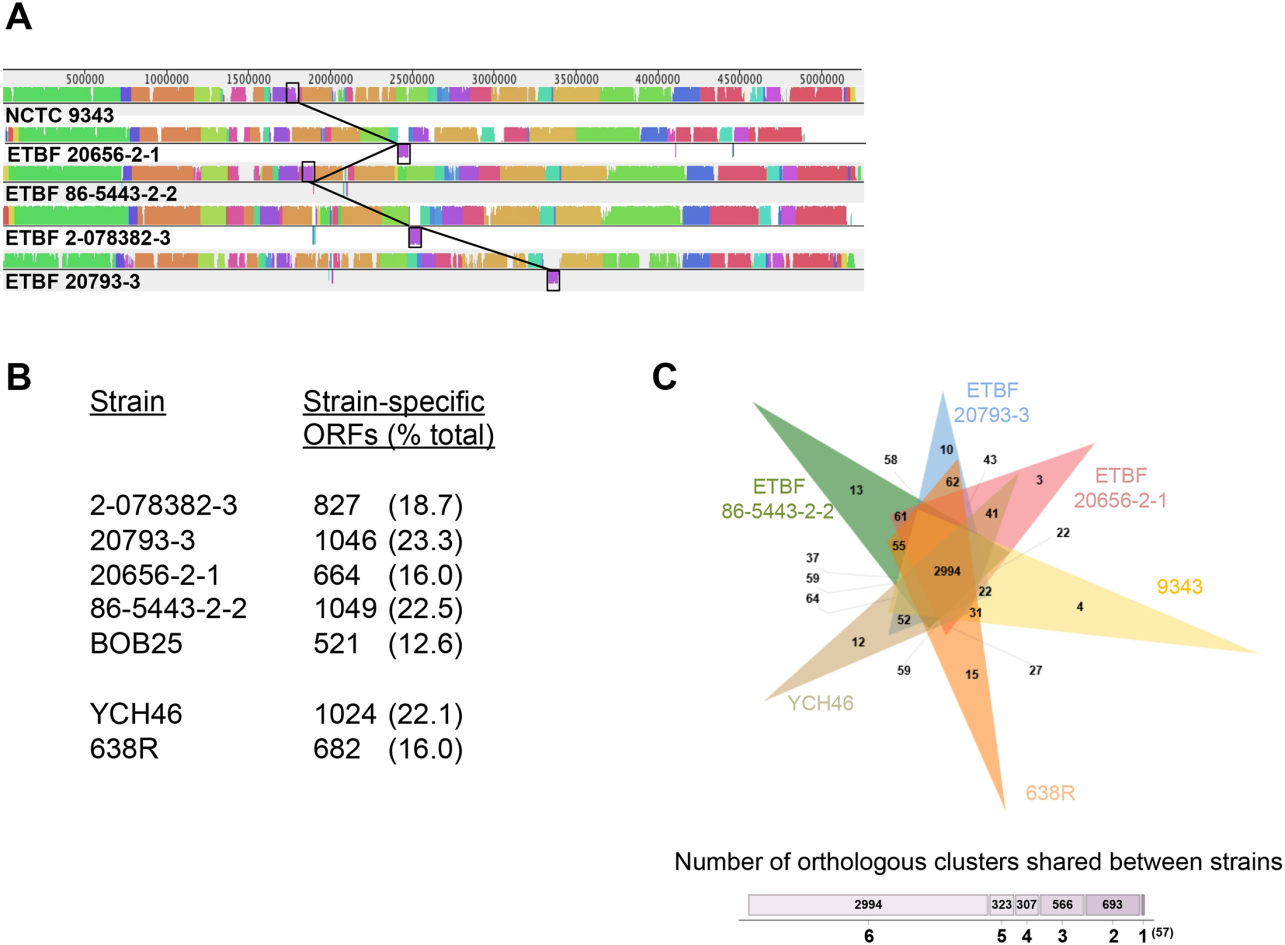
Diversity of *B*. *fragilis* ETBF and NTBF strains. (A) The genome sequences of ETBF strain were aligned to that of NTBF strain NCTC 9343 using Mauve. Blocks of homology between strains are color-coordinated. Non-homologous regions are white, and inverted regions are shown below the center line. Regions containing CTn9343 (which lacks the BFT PAI) and CTn86 are boxed. The diversity of the location of this mobile element is highlighted by interconnecting lines. (B) Intra-species variation is shown by pairwise comparison of non-orthologous coding sequences (<10% identity as determined by BLASTp using CLgenomics software) present in five ETBF and two NTBF strains to NTBF strain NCTC 9343. (C) Orthologous protein clusters observed in three ETBF and three NTBF strains. The Venn diagram indicates overlapping clusters between strains. The total number of clusters that are unique to a single strain and that are shared between 2–6 strains is also shown.

An initial multiple sequence alignment showed that large genomic segments are conserved in all of the ETBF strains and the NTBF reference strain NCTC 9343, but also revealed considerable variation between the ETBF genomes and the NTBF genome (Fig 2A). Contigs were initially reordered using NCTC 9343 as a guide so that inversions or rearrangements within contigs could be identified in the multiple genome alignment. Whole contigs that could not be aligned were examined individually and found to contain either plasmid sequences or no identifiable ORFs (Table 1). Only one copy of the BFT PAI/CTn86 region was identified in each genome by both BLASTp analysis and whole genome alignment. We identified several regions in the ETBF genomes that lack homology to any part of the NCTC 9343 genome. We also observed examples of genomic rearrangement in which specific loci were moved to different chromosomal locations. Based on BLAST searches, ~13–23% of the predicted open reading frames (ORFs) in each of the ETBF genomes are absent from the NTBF 9343 genome (<10% identity) (Fig 2B; S1 Table). The majority of these genes encode proteins of unknown function, but some are predicted to encode proteins involved in capsule formation, metabolism, protein transport and signal transduction. ETBF-specific genes that encode putative toxin-antitoxin systems, cytolysins, restriction modification systems, glycosylases and numerous mobile DNA elements were also identified in each strain (S1 Table). Many of the unique genes were found in clusters, suggesting they form operons that were introduced en bloc via horizontal transfer (perhaps by the aforementioned mobile DNA elements) or duplication events.

A broader analysis indicated that a similar degree of variation is observed when the genomes of any two *B*. *fragilis* strains are compared in a pairwise fashion. About 16–22% of the ORFs present in the genomes of two other NTBF strains, 638R and YCH46, are absent from the NCTC 9343 genome (Fig 2B). An OrthoVenn diagram used to determine the degree of commonality among three ETBF strains and three NTBF strains showed that 2994 orthologous clusters identified based on protein homology were present in all six strains, but that another 1889 clusters were present in 2–5 strains (Fig 2C)[31]. In contrast, a tiny number of clusters (57) were found in only one strain, and no strain contained more than 15 unique clusters. The results suggest that the genomes of both ETBF and NTBF strains are comprised of a large set of core genes that presumably mediate key functions plus a smaller patchwork of ancillary genes that are readily exchanged from one strain to another.

**Fig 3:**
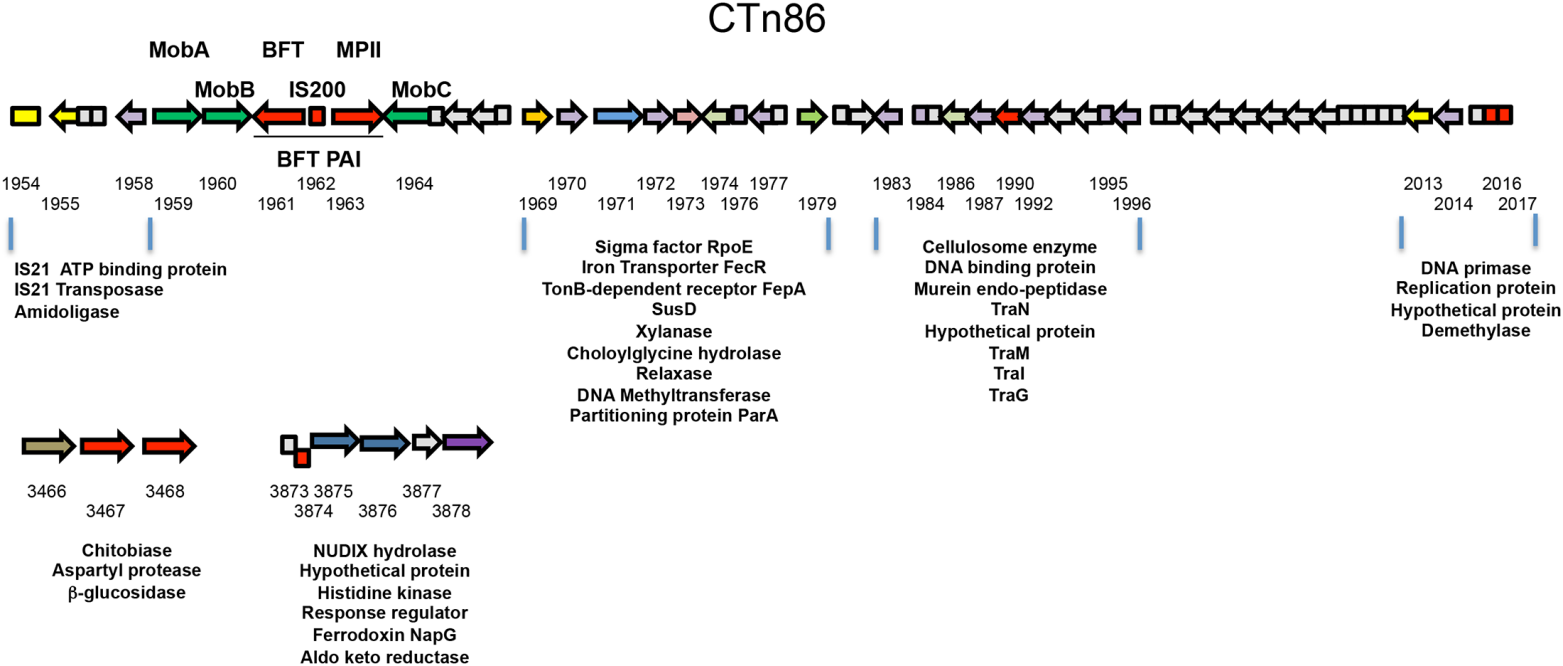
Genes conserved in ETBF strains are located predominantly in the BFT PAI and CTn86. The three regions of the ETBF strain 20656-2-1 genome that contain genes present in all five ETBF strains but not present in the NTBF reference genomes NCTC 9343, 638R, and YCH46 are shown. The conserved genes are shown in red. Other genes that encode proteins with predicted functions are listed and color-coded based on the COG database classification [51] Hypothetical genes are shown in gray. The penultimate four digits of the Genbank accession numbers of genes of known or predicted function are indicated (the final “0” in the accession numbers have been omitted).

Interestingly, BLAST searches showed that only a small group of genes are shared among all five of the EBTF strains but absent from the three reference NTBF strains. The conserved ETBF-specific genes include the Bft gene, a gene encoding a closely related protease (MPII) and several genes present in the CTn86 conjugative transposon (Fig 3, S2 Table). Outside the BFT PAI/CTn86 region, only two genes located within an operon encoding an outer membrane aspartyl protease and a β-glucosidase as well as a hypothetical gene encoding a small protein are conserved. The regions that contain these genes have undergone genomic rearrangement in the NTBF strain NCTC 9343.

While these observations suggest that ETBF strains are distinguished from NTBF strains primarily by the presence of the BFT PAI and closely linked flanking elements, it should be noted that a larger set of ETBF-specific genes (194) that are not present in NCTC 9343 was identified in at least four out of five ETBF strains. Most of these genes encode hypothetical proteins of unknown function, but genes with annotations encoded functions such as metabolism, DNA transfer, capsule biosynthesis, LPS biosynthesis, and transcriptional regulation (S3 Table). It is conceivable that some of these genes play a role in producing phenotypes that distinguish ETBF strains from NTBF strains and that ETBF strains that lack these genes produce compensatory factors. Furthermore, we also identified large prophage regions containing phage, bacterial, and hypothetical genes in each of the ETBF strains that are not present in NTBF strain NCTC 9343 (S4 Table). Three of the strains we sequenced harbor DNA segments that are related to phages 6H and RAP44 and the BOB25 genome also harbors the 6H phage. These phages are associated with distant relatives of the *Bacteroides* called *Flavobacteria* that colonize fish and birds [32, 33]. An ~80 kB segment was also detected in ETBF strain 20793–3 that is only distantly related to known phages but that shares several genes with the Mimivirus *Megavi chiliensis*. BLAST analysis shows that this region encodes proteins that are likely involved in polysaccharide B biosynthesis in *Bacteroides*. Of the three NTBF reference genomes, only strain YCH46 contains the 6H phage, while strain NCTC 9343 does not contain any significant prophage regions. Integration of these prophages may enable ETBF strains to acquire new genes that produce distinctive phenotypes.

The BFT PAIs in the ETBF genomes that we sequenced differ slightly from those that have already been characterized [11, 34]. In all four strains the ~ 6 kb BFT PAI contains a small conserved open reading frame (ORF) that encodes an IS200-like transposase that has not been described previously. This ORF is situated between the *bft* gene and the gene encoding the MPII protease (Fig 3). None of the strains contain ORF 1 found in the BFT PAI of a strain isolated from a lamb [11], and only strain 86-5443-2-2 contained a previously described gene (ORF A) that is located between *mobB* and *bft* [34]. Presumably these two genes are not essential for ETBF survival in the gut. Like previously described BFT PAIs, the BFT PAIs that we sequenced are integrated into the larger conjugative transposon CTn86 and are flanked by the MobA/B to MobC genes (Fig 3). The genome of NTBF strain NCTC 9343 contains a segment that is homologous to the CTn86 element but that lacks the BFT PAI (CTn9343), but this segment is absent from NTBF strain 638R (S1 Fig). Because the regions that flank CTn86 vary widely among the ETBF strains (Fig 2A) it is likely that either this element integrated into different sites in the chromosome or there were large genomic rearrangements following the integration event.

**Fig 4:**
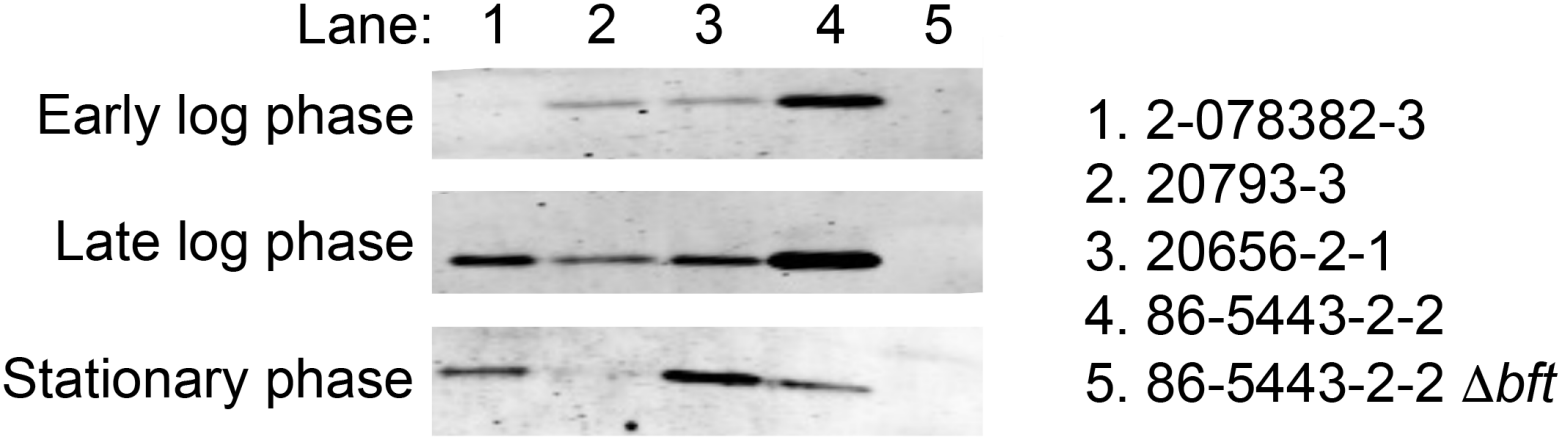
ETBF strains secrete variable amounts of mature Bft. Culture supernatants were obtained from ETBF strains at the indicated growth phases. The volume of each supernatant was normalized based on cell number (OD_600_) and proteins were concentrated 40-fold. Bft was then detected by Western blot using an anti-peptide antiserum.

Although the gene content of the BFT PAIs in the four strains is very similar, the level of mature Bft produced by each ETBF strain differed considerably. Western blot analysis of culture supernatants showed that strain 86-5443-2-2 secreted the most Bft, strains 2-078362-3 and 20656-2-1 secreted intermediate amounts, and strain 20793–3 secreted the least toxin (Fig 4). The level of Bft peaked at late log phase, which suggests that the protein is produced primarily during active growth. Our data are consistent with the results of previous studies showing that ETBF strains exhibit variable toxicity against HT-29 tissue culture cells and that strain 86-5443-2-2 is highly toxigenic [35]. Because strains that produce the same isoform of Bft secrete both high and low amounts of the protein (based on the nucleotide sequence of the *bft* gene all of the strains produce isoform 2 except strain 20656-2-1, which produces isoform 1), there was no correlation between the protein sequence and the level of secretion. It remains to be determined whether disparities in the accumulation of the mature toxin result from differences in transcription, translation, secretion efficiency or proteolytic maturation.

### NTBF strain NCTC 9343 secretes proteins that inhibit the growth of ETBF strains

**Fig 5:**
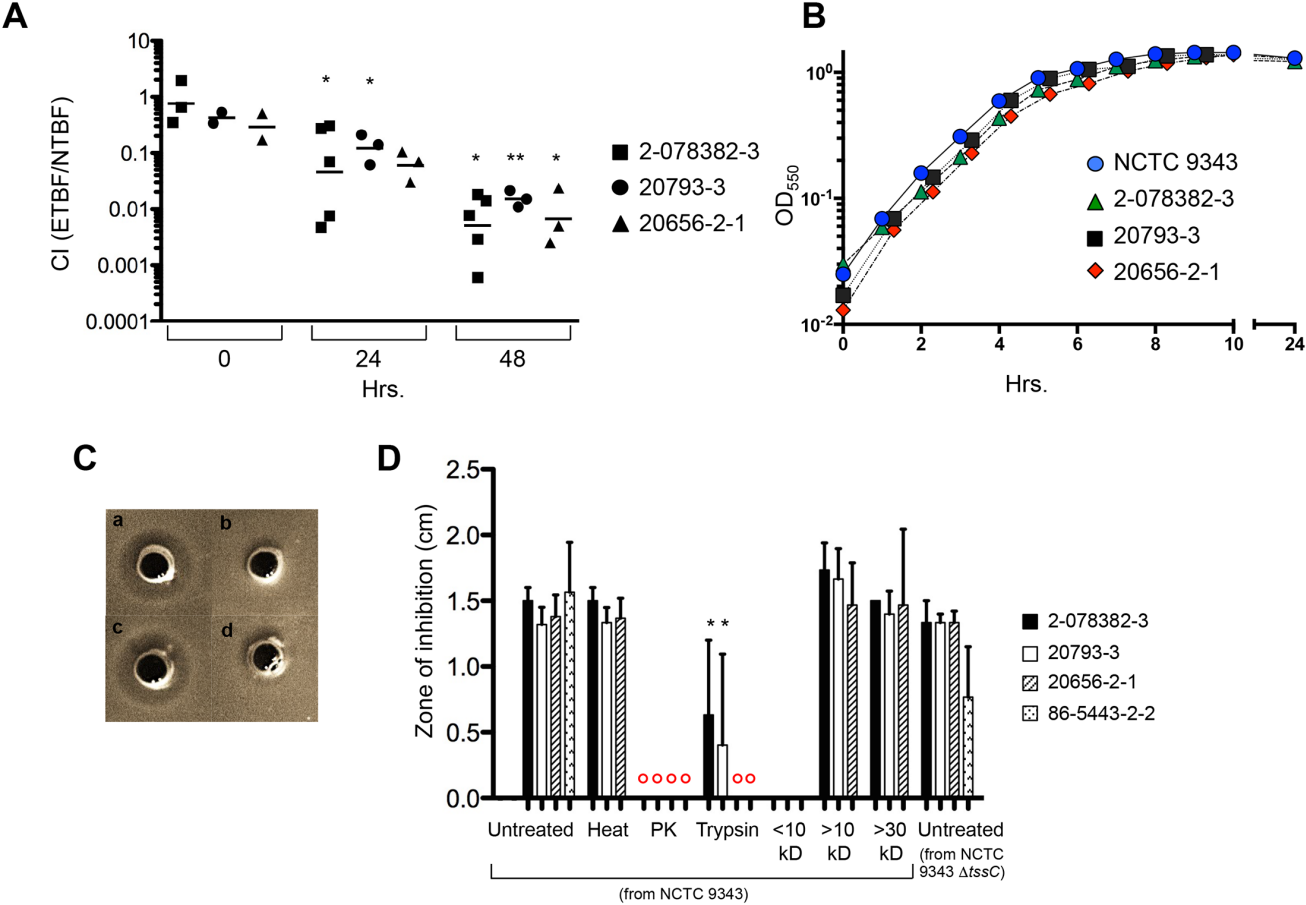
NTBF strain NCTC 9343 outcompetes ETBF strains and secretes a growth-inhibitory protein. (A) *In vitro* competition assay. Strain NCTC 9343 was co-cultivated with one ETBF strain and passaged after 24 h. The competitive index (CI, ratio of ETBF/NCTC 9343 DNA) was determined by strain-specific qPCR. Significance was determined by ANOVA across all time points for each strain (2-078382-3, p = 0.016; 20793–3, p = 0.0048; 20656-2-1, p = 0.048). Tukey’s multiple comparison test results are shown comparing 24 and 48 h time points to the inoculum (* = p<0.05, ** = p<0.01). Each data point is an independent measurement. (B) Growth curve of NCTC 9343 and individual ETBF strains. One representative experiment is shown. (C) Agar diffusion assay. Supernatant obtained from a culture of strain NCTC 9343 was untreated (a) PK treated (b), heat treated (c), or trypsin treated (d) and then added to wells in plates seeded with the ETBF indicator strain 2-078382-3: (D) Quantitation of agar diffusion assays in which culture supernatants from strain NCTC 9343 WT or NCTC 9343 Δ*tssC* were added to ETBF strain 2-078382-3, 20793–3, 20656-2-1, or 86-5443-2-2. Supernatants were untreated, heat-treated, protease treated, or fractionated by molecular weight. A Student’s t-test was used to demonstrate a significant loss of activity (*, p<0.05). The red circles indicate a complete loss of activity.

Bacteria must be able to compete with other organisms in order to survive in a microbiome. For this reason we wished to determine whether the multitude of genes that are unique to the ETBF strains or to strain NCTC 9343 might confer a fitness advantage when they are co-cultivated. Cells from one of the ETBF strains were incubated with an equal number of NTBF 9343 cells in laboratory broth and the relative cell number was monitored at different time points by qPCR using strain-specific primers (S2 Fig). We found that at 24 h, NCTC 9343 outnumbered cells from strains 2-078382-3, 20793–3, 20656-2-1 by 5–10 fold (Fig 5A). By 48 h, the difference had risen to ~100-fold [corresponding to a ETBF/NTBF competitive index (CI) of ~0.01]. No difference in growth rate was detected when each strain was grown individually in broth culture (Fig 5B). These results imply that NCTC 9343 either utilizes the resources of the growth medium more effectively than the ETBF strains or produces molecules that actively inhibit their growth.

Because *B*. *fragilis* has been shown to secrete anti-microbial molecules that target closely related organisms [18, 36, 37], we hypothesized that NCTC 9343 might use a similar strategy to gain a competitive advantage over the ETBF strains. To explore this possibility, we isolated culture supernatants from all of the strains and tested them for the presence of growth inhibitory activities using an agar diffusion assay. Interestingly, we found that the culture supernatant from strain NCTC 9343 inhibited the growth of all four ETBF strains (Fig 5C and 5D). The active molecule(s) secreted by NCTC 9343 was heat-resistant but susceptible to both proteinase K (PK) and trypsin digestion (Fig 5C and 5D). Incubation of cells with PK-treated medium alone had no effect on growth (data not shown). These results implied that one or more peptides or proteins inhibit the growth of the ETBF strains. Fractionation of the supernatant by ultrafiltration suggested that the inhibitory activity is associated with at least one protein that is >10 kD. Although strain NCTC 9343 has been shown to secrete effectors that inhibit the growth of other *Bacteroides* through a T6SS [20], elimination of a core component of the secretion machinery (TssC) did not affect its ability to inhibit the growth of the ETBF strains (Fig 5D). Unlike the NTBF strain, the ETBF strains did not consistently produce an anti-microbial factor that was active against either NCTC 9343 or other ETBF strains (data not shown). In addition, the factor produced by NCTC 9343 did not inhibit its own growth or that of NTBF strain 638R.

Although the ETBF strains did not exhibit anti-microbial activity against the *B*. *fragilis* strains tested here, our genomic analysis suggests that they may produce factors that inhibit the growth of other bacteria. Using the program BAGEL, which searches for the conservation of both protein domains and genomic context, we identified several loci that encode putative secreted small bacteriocins (S3 Fig). The genomes of ETBF strains 2-078382-3 and 86-5443-2-2 appear to encode peptides that belong to the sactipeptide and lanthipeptide classes of bacteriocins that are characterized by the presence of thioether bonds. The genes are found in loci that also encode enzymes involved in peptide biosynthesis and modification (LanB and LanC) and transport (HlyD and ABC transporters). A gene that encodes a putative glycocin (a peptide modified by a lipid or carbohydrate moiety) is located in a region of the 20793–3 genome that likewise encodes several genes involved in peptide transport. These classes of bacteriocins have been described mainly in Gram-positive bacteria, but a sactibiotic gene cluster was recently identified in *Bacteroides* sp. 3_1_19 through an *in silico* analysis [38].

We also identified T6SS loci in all of the ETBF genomes, although the locus in strain 20793–3 lacks most of the genes (S4A Fig). The T6SS locus in strain 2-078382-3 resembles those found in the NTBF strains NCTC 9343 and 638R. Because there are several non-homologous regions, however, it seems likely that selective pressures operating on each of the strains have led to continuing evolution of the locus. Interestingly, the T6SS locus in ETBF strain 20656-2-1 is very similar to the corresponding locus in *B*. *cellulosyliticus*. This observation is consistent with other evidence that T6SS loci can be exchanged between species via horizontal gene transfer [24]. Western blot analysis of culture supernatants using an antibody against one of the major T6SS components (TssD) confirmed that the locus was expressed under laboratory growth conditions by all of the strains except 20793–3, albeit at a much lower level than the NTBF strains 9343 and 638R (S4B Fig).

### ETBF strains exhibit greater biofilm activity than NCTC 9343

**Fig 6:**
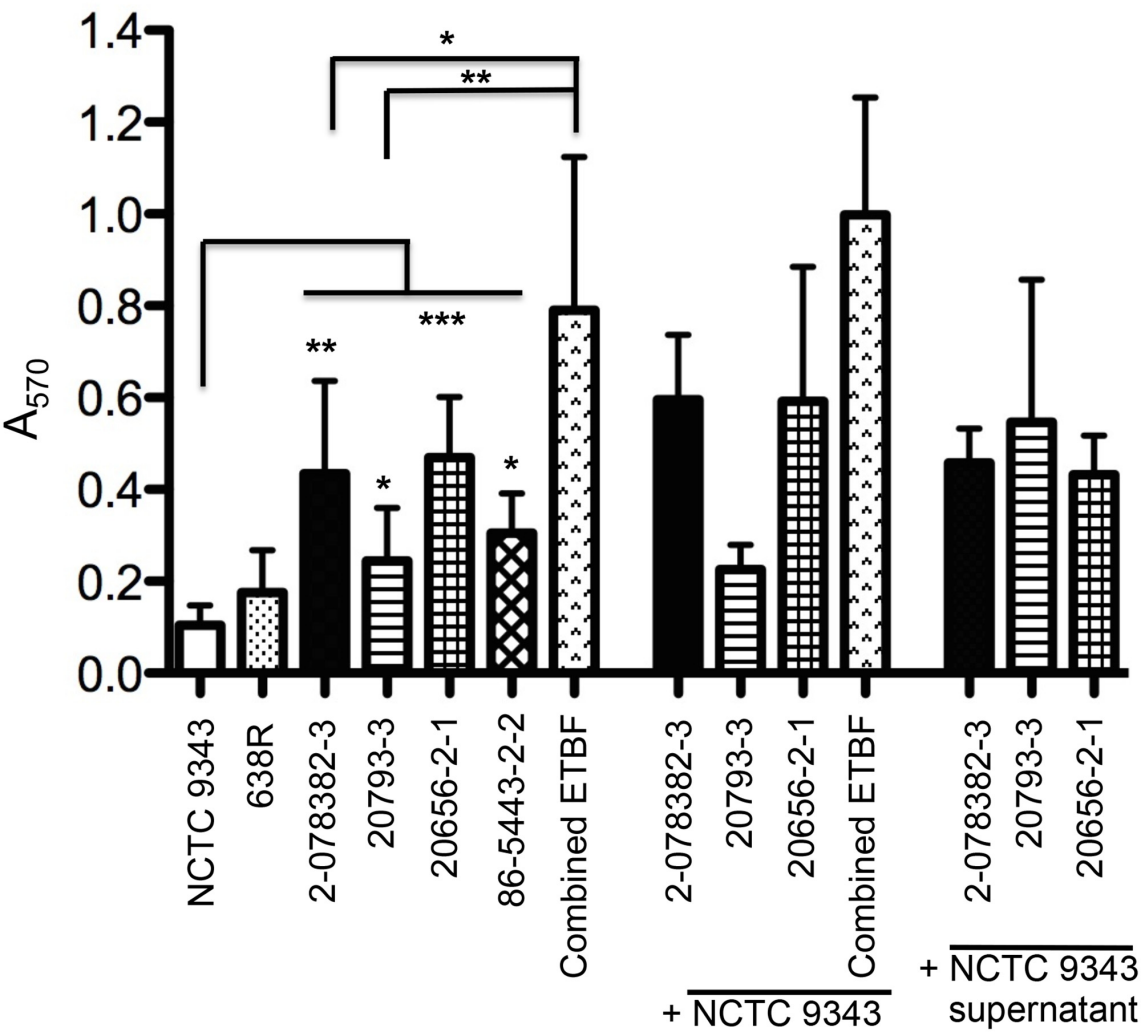
ETBF strains exhibit enhanced biofilm formation. (A) *B*. *fragilis* strains forming biofilms in microtiter plates were stained with crystal violet and quantified at A_570_. A single strain or a combination of three ETBF strains (2-078382-3, 20793–3, and 20656-2-1) was grown in BHIS medium, in co-culture with strain NCTC 9343, or in an equal mixture of BHIS medium and NCTC 9343 culture supernatant. Data from at least three independent experiments are shown along with the standard deviations. Asterisks indicate significant changes as determined by unpaired t-test: * p<0.05, **p<0.01, ***p<0.001.

Bacteria that reside in the gut, including *Bacteroides*, are capable of forming biofilms in association with both luminal particulates and the colonic mucosa [27, 39]. Because the formation of a biofilm can significantly affect the interactions of a bacterial species with both other microorganisms and the host, we wished to determine whether the ETBF strains differ from NTBF strains in their ability to form a biofilm. In our experiments we detected biofilm formation by staining cells that adhered to microtiter plates with crystal violet [40]. Consistent with previous results [25], we found that NCTC 9343 produced weak biofilms after growth in rich medium (Fig 6). NTBF strain 638R also exhibited a low level of biofilm activity. ETBF strains 2-078832-3, 20793–2, 20656-2-1, and 86-5443-2-2, however, all showed a markedly greater tendency to form biofilms. Furthermore, when three of the ETBF strains were co-inoculated, they exhibited a higher level of biofilm production than when they were grown individually. Deletion of *bft* from strain 86-5443-2-2 did not decrease biofilm formation, suggesting that the toxin itself is not required for this phenotype (data not shown). We also found that whereas NCTC 9343 inhibited the growth of the three ETBF strains in broth culture, incubation of either NCTC 9343 cells or NCTC 9343 culture supernatant with the ETBF strains did not significantly affect their ability to form biofilms. While this observation may seem paradoxical, it is consistent with the results of previous studies that have shown that bacteria living in biofilms are hyperresistant to antibiotic treatment due to both an increase in the expression of resistance mechanisms and a decrease in the diffusion of the anti-microbial agents. Interestingly, the ETBF strains possess the genes for a polyamine biosynthetic pathway that has been associated with biofilms and colorectal cancer [41] and that is required for biofilm formation in *Vibrio* species [42]. The combination of increased biofilm formation and the production of secondary metabolites and Bft may increase the ability of ETBF strains to cause disease.

## Discussion

By determining the genome sequence of four different ETBF strains and by studying their growth properties both in mixed cultures and in monocultures, we obtained evidence that these strains constitute a genetically diverse group that nevertheless share common phenotypes. We found that the genomes of the ETBF strains vary to about the same degree as the genomes of NTBF strains and are distinguished from NTBF strains mainly by the presence of the BFT PAI and specific elements of CTn86. The ETBF strains do not form a phylogenetic cluster, but instead appear to have emerged in multiple independent events. Indeed the transfer of the BFT PAI may be facilitated by its location within CTn86, a transposable element whose heterogeneous location in *B*. *fragilis* genomes suggests that it is highly mobile. Despite their genetic diversity, the ETBF strains exhibited similar growth properties. All of the ETBF strains we tested displayed reduced competitiveness in mixed cultures with NCTC 9343 and were subject to the action of an anti-microbial factor secreted by the NTBF strain. In contrast, the ETBF strains produced biofilms more readily than NCTC 9343. Taken together, the results suggest the existence of a recurring selection process in which the horizontal transfer of the BFT PAI and other genetic elements into an NTBF genome ultimately leads to the evolution of a phenotypically distinct subpopulation. The evolution of the strains we analyzed might have been accelerated by the introduction of a wide array of putative mobile DNA elements that include several prophage regions that are not associated with previously described phage genomes.

Several lines of evidence suggest that the acquisition of novel genetic elements enable ETBF strains to occupy a different niche from NTBF strains. First, a significant fraction of the genes found exclusively in the genomes of three of the four strains we sequenced are predicted to function in metabolism (e.g., glycosyltransferases), membrane processes or cell wall/capsule biosynthesis. A previous analysis of the genomes of different *Bacteroides* species suggested that the ability to utilize different nutrient resources likely affects habitat adaptation [43]. Furthermore, changes in the capsule or cell surface have been shown to affect immune activation [1] and may also affect interactions with the mucosal layer. Second, the enhanced ability to form biofilms may promote preferential growth in specific sites within the colon. Finally, the sensitivity of the ETBF strains to an antimicrobial protein produced by NCTC 9343 suggests that they may not be able to coexist in the same spatial environment. Indeed the presence of closely related strains exerts a strong selective pressure on the survival of *Bacteroides* in a mouse model [44]. The observation that the ETBF strains lose their susceptibility to the NTBF antimicrobial factor once they form a biofilm, however, raises the possibility that biofilm formation evolved as a protective mechanism. A recent mathematical model also showed that communities of antibiotic producers and antibiotic-sensitive organisms can be stabilized by the degradation of the antibiotic by a third organism [45]. Thus in the context of the entire gut microbiome, ETBF strains may coexist with NTBF strains more effectively than they do in head-to-head competition experiments.

The discovery that NCTC 9343 secretes a protein that inhibits the growth of ETBF strains is consistent with other evidence that the *Bacteroides* actively produce antimicrobial factors that target closely related organisms. Indeed a variety of recent studies have demonstrated that NTBF strains exhibit a strong tendency to produce factors that inhibit the growth of other NTBF strains. One study showed that the growth of NCTC 9343 and other NTBF strains was inhibited by a putative pore forming protein (termed BSAP-1) produced by strain 638R [18]. Several other studies have shown that NTBF strains (including NCTC 9343) use T6SSs to secrete factors that inhibit the growth of other *Bacteroides* [20, 22, 23]. The finding that the inhibitory factor we identified is not secreted by the T6SS confirms that NCTC 9343 uses multiple mechanisms to suppress the growth of distinct competitors. Our genomic and experimental analysis suggest that ETBF strains may rely less on T6SSs than NTBF strains but may use antimicrobial peptides to inhibit the growth of other organisms. The remarkably diverse array of parallel antimicrobial systems that appear to be used by *B*. *fragilis* suggests that there is intense competition for limited resources in the gut that leads to an accumulation of armaments to block the growth of siblings and a selection for variants that can thrive in alternative niches.

Perhaps most significantly, our genomic characterization of ETBF strains provides evidence that the diversity of the human gut microbiome has been seriously underestimated. During the last several years many studies have been published that characterize changes in the microbiome that occur in disease states or in response to various stimuli that classify organisms based on the sequencing of 16S rRNA genes. While this method is highly useful to identify global changes, it does not provide an effective means to detect changes at the strain level that may nevertheless have important biological consequences. In this regard it should be noted that a recent study showed that only a discrete subset of *B*. *fragilis* strains obtained from patients with inflammatory bowel syndrome stimulate high levels of IgA production [46]. Especially in light of our genomic analysis, the observation that ETBF strains secrete different amounts of Bft raises the possibility that they differ considerably in their ability to cause disease. Thus from a clinical perspective, it may ultimately be useful to subdivide ETBF strains into distinct subtypes.

Finally, our analysis of ETBF genomes suggests that evolution within the microbiome probably does not often occur through a linear diversification process, which may be rather slow, but rather through the acquisition of multiple genes via horizontal transfer from a variety of related organisms. Presumably this fast-track evolutionary strategy is necessary because the acquisition of a single gene or PAI is often insufficient to support growth in a new niche. Indeed, to explain the contribution of the great variety of strains that co-exist in the gut microbiome to health and disease, it may be necessary to elucidate the role of a multitude of genes in promoting adaptation to the colonic environment.

## Materials and Methods

### Bacterial strains and media

*B*. *fragilis* strains NCTC 9343, 2-078382-3, 20793–3, and 20656-2-1 were obtained from the ATCC. Strain 86-5443-2-2 and the isogenic *bft* deletion strain were obtained from Dr. Cindy Sears (Johns Hopkins University). The NCTC 9343 Δ*tssC* strain [20] was obtained from Dr. Andrew Goodman (Yale University). All strains were grown in BHIS medium supplemented with 10 μg/ml erythromycin as needed at 37°C in anaerobic jars or test tubes. Growth curves were constructed by diluting overnight cultures 1:50 and measuring OD_550_ over time.

### Genome sequencing and comparative genomics

Genomic DNA from strains 2-078382-3, 20793–3, 20656-2-1 and 86-5443-2-2 was isolated using the Wizard DNA isolation kit (Promega). Genome library preparation, sequencing, and bioinformatics were performed in conjunction with the NIDDK Genomics Core Facility (Bethesda, MD) for strain 2-078382-3 and ChunLab (Seoul, Korea) for all the other strains. Genomic DNA libraries were prepared for paired-end sequencing with inserts of either 500 bp or 300 bp using an Illumina MiSeq sequencer. Sequences were annotated using RAST software and software developed by ChunLab. Genome comparisons were performed using Mauve [47], RAST/SEED server [48], ResFinder [49], BAGEL [50], PHAST [51] and CLGenomics software. The genome sequences have been deposited at GenBank under the accession numbers LIDV00000000 (2-078382-3), LIDT00000000 (20793–3), LIDU00000000 (20656-2-1), and LIDS00000000 (86-5443-2-2). The versions of the genome sequences described in this paper are LIDV/LIDT/LIDU/LIDS 01000000.

### Competition assays

Overnight cultures in BHIS were diluted 1:20 and grown to OD_600_ = 0.1–0.2 prior to mixing at a 1:1 ratio. Cultures were sampled immediately after inoculation and after 24 h. The mixed cultures were then further diluted at a 1:50 ratio and sampled 24 h later (48 h time point). Genomic DNA was isolated from each sample. Strain-specific-primers that detect *bft* (BFT RT-PCR F2 5’-GTTCAACGGCAGGGACAAGGAT-3’, BFT RT-PCR R2 5’-TGCGATAAAAACGTAATACTGCGAACTCAT-3’ and *nanH2* (NanH2 F1 5’-GGGCGGAAAGTGATACTGAATGCTAAT-3’, NanH2 R1 5’-GGCGTCCCCCTACTCCTACCCTATGA-3’) were used to measure the relative proportion of each strain using qPCR with POWER SYBR green PCR Mastermix (Applied Biosystems) and a Bio-Rad C1000 Touch ThermoCycler. Statistics were generated by ANOVA to determine changes over time for each strain with Tukey’s post-test to determine pairwise comparisons.

### Growth inhibition assays

Indicator strains were grown overnight in BHIS and then inoculated into BHIS-0.75% agar at a 1% v/v ratio (adapted from [52]). After the agar solidified, wells were cut into the agar using the wide end of a Pasteur pipette (5 cm). Producer strains were grown in parallel in 10 ml overnight cultures. The cultures were centrifuged (4,000 x g, 20 min, 4°C) and residual cells were removed from the supernatant by filter sterilization using a 0.45 μm filter. Portions of the culture supernatants were then heat treated (95°C, 10 min) or enzymatically treated at 37°C for 1 h with PK (1 mg/ml final concentration) or trypsin (0.1 mg/ml) and heat inactivated. Subsequently 100 μl of the treated or untreated supernatants were added to the wells, and the plates were incubated overnight at 37°C. The zone of inhibition was determined by measuring the diameter of the clear agar surrounding each well. Significant differences for non-zero values were determined using a Student’s t-test.

### Biofilm assays

Biofilm assays were performed as previously described [25]. Briefly, cells were grown overnight and diluted to OD_600_ = 0.08–0.1. Aliquots (20 μl) of cells were then added to 180 μl of BHIS and loaded into 96-well polystyrene tissue culture treated plates (Costar) in triplicate. Some samples contained cells that were inoculated into BHIS containing 50% filter-sterilized NCTC 9343 culture supernatant or contained a 1:1 mixture of ETBF and NTBF strains (10 μl of each). As a control, BHIS alone was added to several wells. Plates were incubated for 24 h at 37°C. Subsequently the contents of each well were removed by aspiration and the wells were washed three times with 100 μl PBS. The plates were then dried at 65°C for 30 min and cells that remained in each well were stained with 1% crystal violet for 5 min. The wells were then rewashed as described above and the plates were dried at 65°C for another 10 min. The absorbed crystal violet was solubilized in 150 μl 30% acetic acid and measured in a spectrophotometer at 570 nm. Pairwise statistics were performed using a Student’s t test.

### Detection of Bft and TssD in culture supernatants

Cells were grown to early log (OD_600_ = 0.5), late log (OD_600_ = 1.0) or stationary phase (24hrs) and then centrifuged twice (4000 x g, 15 min, 4°C). Proteins were precipitated from culture supernatants with 10% TCA and washed twice with cold acetone before resuspension in SDS-PAGE sample buffer. Proteins were resolved on 8–16% SDS-PAGE minigels and transferred to nitrocellulose using an iBlot device (Life Technologies). Western blotting was performed using polyclonal rabbit antisera directed against a C-terminal peptide of Bft (NH_2_-CSEKNMDIIAKNLGWEAADGD-COOH) or full-length His- tagged TssD. Antibody-antigen complexes were detected using a fluorescently labeled goat anti-rabbit antibody and an Odyssey near-infrared imaging system (Licor). To make the Tss protein, the *tssD* gene (BF9343_1943) was cloned into pET28b and expressed in *E*. *coli* BL21(DE3) harboring pRARE. Inclusion bodies enriched in TssD were solubilized in 8M urea and the protein was purified on Ni-NTA agarose (Qiagen). The protein was then further purified by SDS-PAGE. The band containing TssD was excised from the gel and used to immunize rabbits.

## Supporting Information

S1 Fig Alignment of the BFT PAI and larger mobile element Ctn86/Ctn9343. (TIF)

S2 Fig Strain-specific primers for quantitative PCR. (TIF)

S3 Fig Identification of loci encoding putative bacteriocins in ETBF strains. (TIF)

S4 Fig Diversity of T6SS loci among *B*. *fragilis* strains. (TIF)

S1 Table List of genes present in ETBF strains but not NCTC 9343 with annotations. (XLSX)

S2 Table Conserved ETBF-specific genes. (XLSX)

S3 Table Genes identified in at least four of the five sequenced ETBF strains. (XLSX)

S4 Table Prophage regions identified in *B*. *fragilis* strains. (TIF)
