# Supplementary figures and images for: Genomic Diversity of Enterotoxigenic Strains of Bacteroides fragilis
Source: PLoS One. 2016 Jun 27;11(6):e0158171. doi: 10.1371/journal.pone.0158171 (PMC4922554; doi:10.1371/journal.pone.0158171)

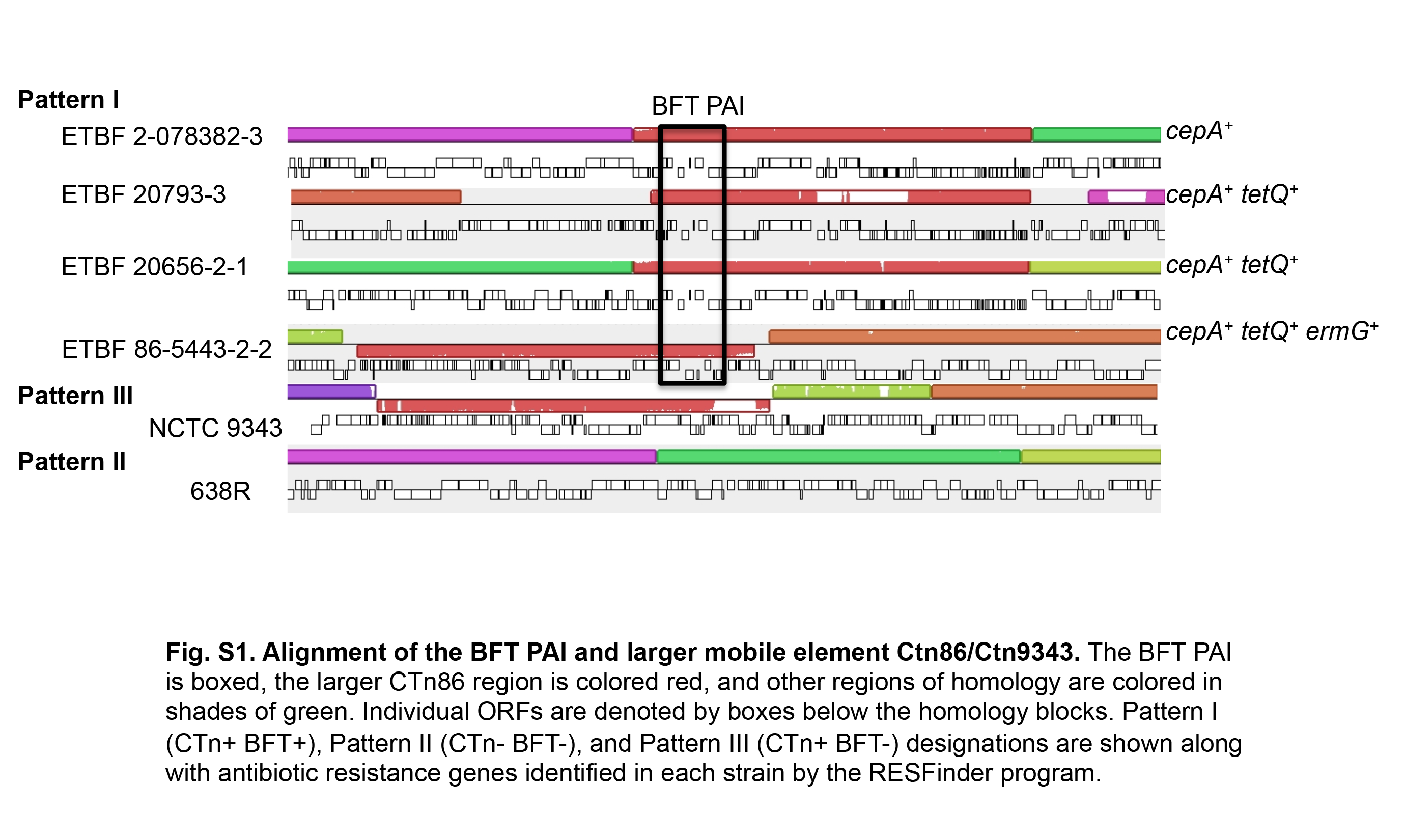

Supplement: S1 Fig — (TIF) [file pone.0158171.s001.tif]

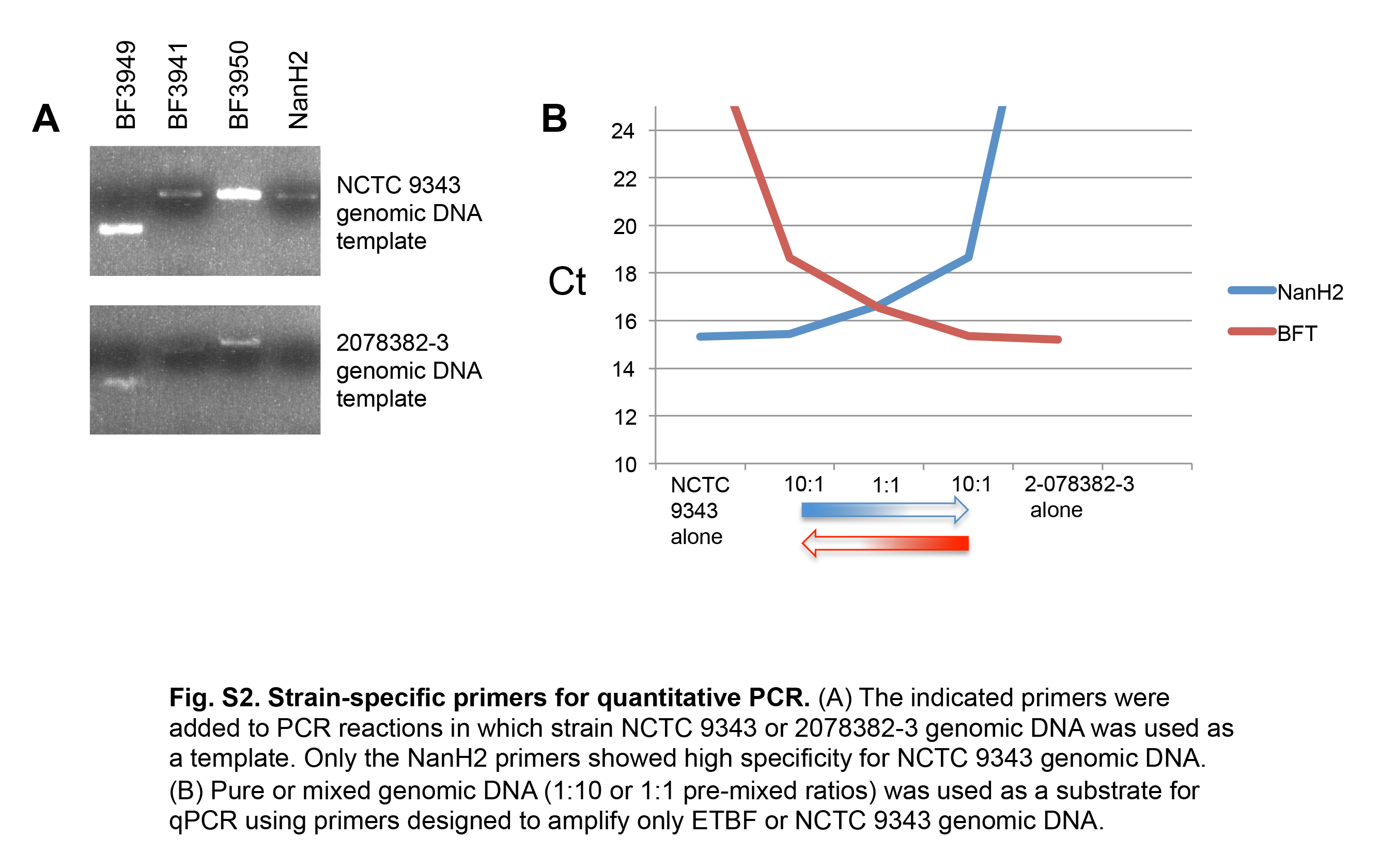

Supplement: S2 Fig — (TIF) [file pone.0158171.s002.tif]

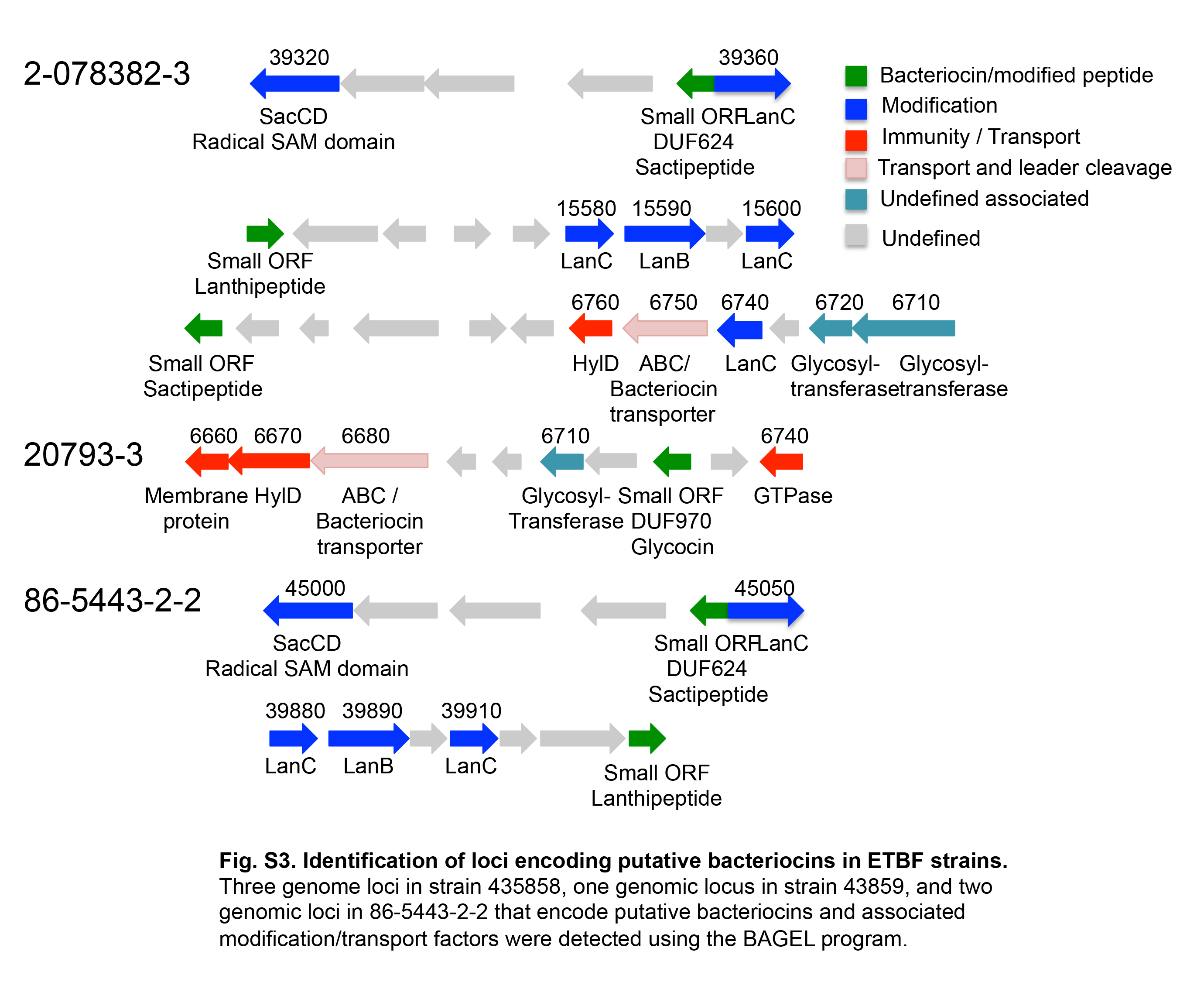

Supplement: S3 Fig — (TIF) [file pone.0158171.s003.tif]

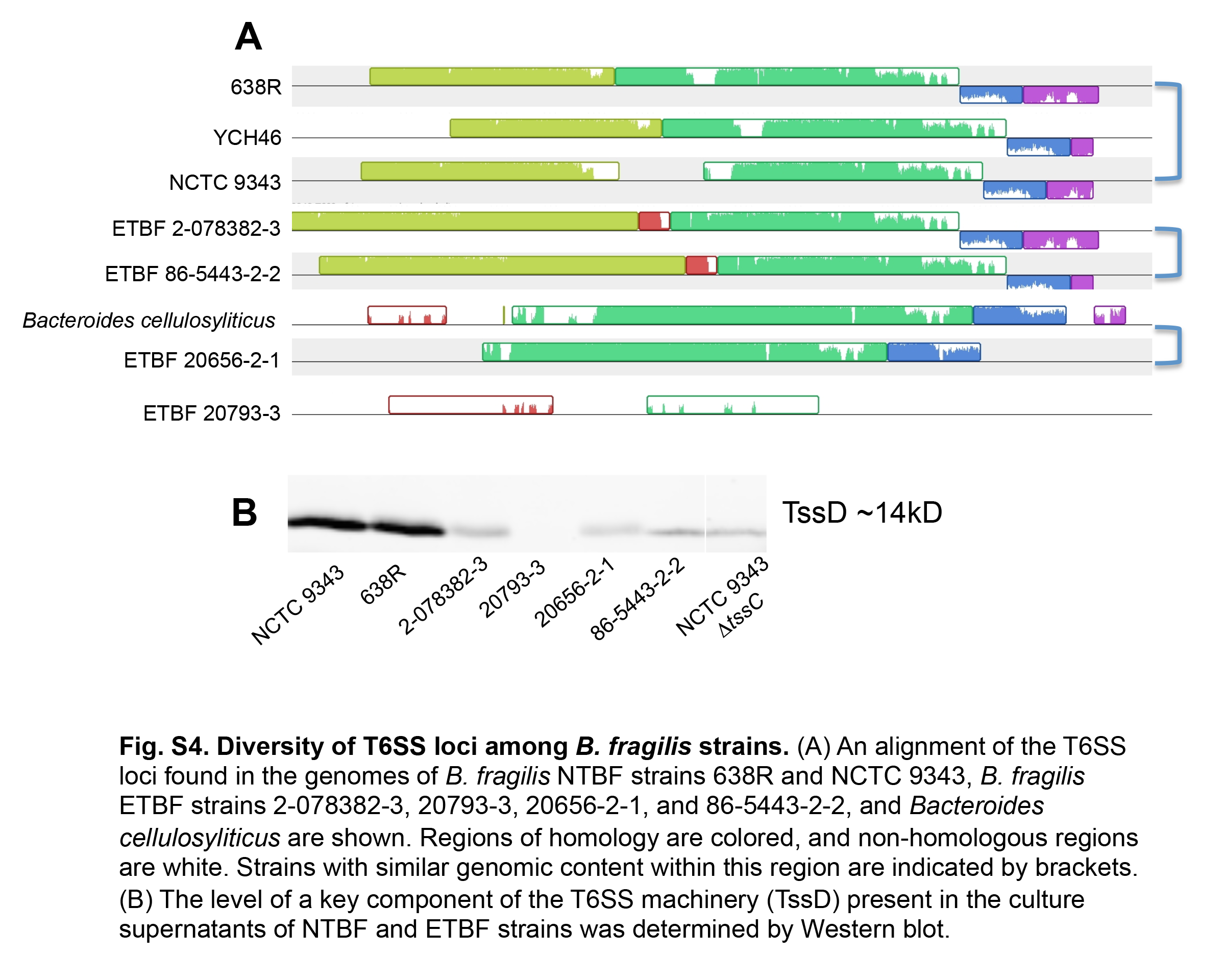

Supplement: S4 Fig — (TIF) [file pone.0158171.s004.tif]

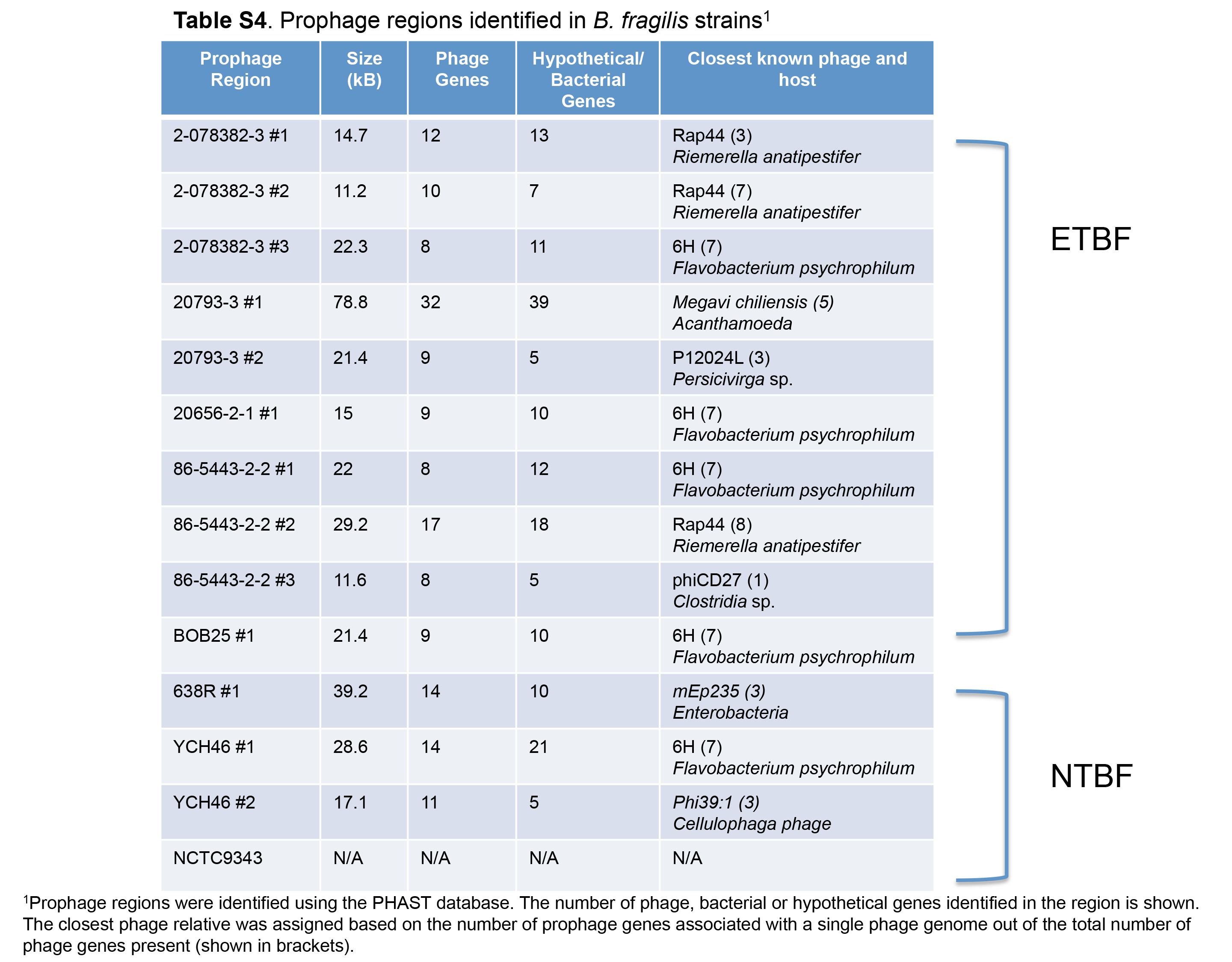

Supplement: S4 Table — (TIF) [file pone.0158171.s008.tif]
